# Structural insight into antibody-mediated antagonism of the Glucagon-like peptide-1 Receptor

**DOI:** 10.1038/srep26236

**Published:** 2016-05-19

**Authors:** Stephanie Hennen, János T. Kodra, Vladyslav Soroka, Berit O. Krogh, Xiaoai Wu, Peter Kaastrup, Cathrine Ørskov, Sif G. Rønn, Gerd Schluckebier, Silvia Barbateskovic, Prafull S. Gandhi, Steffen Reedtz-Runge

**Affiliations:** 1 Incretin Biology, Novo Nordisk, Novo Nordisk Park, DK-2760, Måløv, Denmark; 2 Protein & Peptide Chemistry 3, Novo Nordisk, Novo Nordisk Park, DK-2760, Måløv, Denmark; 3 Protein Structure, Novo Nordisk, Novo Nordisk Park, DK-2760, Måløv, Denmark; 4 Yeast Expression Systems, Novo Nordisk, Novo Nordisk Park, DK-2760, Måløv, Denmark; 5 Protein Chemistry 1, Novo Nordisk A/S, Novo Nordisk China R&D, Beijing, China; 6 Antibody Technology, Novo Nordisk, Novo Nordisk Park, DK-2760, Måløv, Denmark; 7 Protein Interaction, Novo Nordisk A/S, Novo Nordisk Park, DK-2760, Måløv, Denmark

## Abstract

The Glucagon-like peptide-1 receptor (GLP-1R) is a member of the class B G protein-coupled receptor (GPCR) family and a well-established target for the treatment of type 2 diabetes. The N-terminal extracellular domain (ECD) of GLP-1R is important for GLP-1 binding and the crystal structure of the GLP-1/ECD complex was reported previously. The first structure of a class B GPCR transmembrane (TM) domain was solved recently, but the full length receptor structure is still not well understood. Here we describe the molecular details of antibody-mediated antagonism of the GLP-1R using both *in vitro* pharmacology and x-ray crystallography. We showed that the antibody Fab fragment (Fab 3F52) blocked the GLP-1 binding site of the ECD directly and thereby acts as a competitive antagonist of native GLP-1. Interestingly, Fab 3F52 also blocked a short peptide agonist believed to engage primarily the transmembrane and extracellular loop region of GLP-1R, whereas functionality of an allosteric small-molecule agonist was not inhibited. This study has implications for the structural understanding of the GLP-1R and related class B GPCRs, which is important for the development of new and improved therapeutics targeting these receptors.

The glucagon-like peptide-1 (GLP-1) receptor is a class B GPCR and activation by GLP-1 leads to intracellular signalling mediated primarily by the G protein Gαs and subsequent increase of cAMP production^1^. It is well established that activation of GLP-1R in pancreatic beta cells results in glucose-dependent potentiation of insulin secretion and a subsequent decrease of the blood-glucose level^2^. This effect is preserved in patients with type-2 diabetes and a number of GLP-1-based therapies are approved or in late stage clinical trials for treatment of this disease^2^,^3^.

The signature of class B GPCRs is a ~15 kDa N-terminal extracellular domain (ECD) essential for binding to the C-terminal part of the cognate peptide hormones. This particular interaction has been described in molecular details by both NMR spectroscopy and X-ray crystallography using recombinant isolated ECDs^4^,^5^,^6^,^7^,^8^. The N-terminal part of the peptide hormones is essential for activation and competitive antagonists were generated by modifications or deletions of a few amino acid residues^9^,^10^,^11^,^12^. Accordingly, the two-domain ligand binding model suggests that the N-terminal part of the peptide hormones engage the TM and ECL region of the receptor leading to activation and signal transduction^13^. Ligand-receptor crosslinking and mutagenesis have been applied to Class B GPCRs in order to map the interaction of the peptide N-terminus with the binding site of the TM domain^14^,^15^,^16^,^17^. C-terminal truncation of these peptide hormones results in a significant loss of affinity and non-natural modifications are necessary to increase the activity of short peptide agonists^18^,^19^. The glucagon receptor and corticotropin-releasing factor receptor 1 TM domain structures were solved by x-ray crystallography showing the expected topology of the seven transmembrane α-helices^20^,^21^. However the spatial relationship of the ECD and TM domain is not well understood, because the structures were solved separately. An elongated conformation of the ECD and TM domain was suggested recently based on electron microscopy (EM) of an antibody-bound full length glucagon receptor (GCGR)^22^. This conformation may also be representative of the peptide agonist conformation of GCGR and other class B GPCRs, although previous models of GLP-1R suggested a more tilted conformation of the ECD relative to the plane of the membrane in the GLP-1-bound state^23^,^24^.

We recently isolated a new monoclonal anti-GLP-1R antibody (mAb 3F52) by immunization of GLP-1R knock-out mice with the isolated human GLP-1R ECD^25^. High resolution cellular localization of the GLP-1R in monkey pancreas, gastrointestinal, cardiac and renal tissues was revealed by immunohistochemistry using the mAb 3F52, and importantly the specificity was proven by use of *in situ* ligand binding showing the same expression pattern. This antibody seems currently to be the only antibody that measures the GLP-1R expression correctly and specifically^25^,^26^,^27^. In the present work we provide further evidence supporting the high GLP-1R specificity of this antibody.

Antibody Fab fragments are useful tools for structural characterization of target proteins and were used recently to obtain the first crystal structure of the glucagon receptor ECD and a new crystal structure of the glucose-dependent insulinotropic polypeptide (GIP) receptor ECD^28^,^29^. This study describes the crystal structure of the human GLP-1R ECD in complex with Fab 3F52 and reveals the molecular details of antagonism and receptor specificity of Fab 3F52. Interestingly, the inhibitory effect of Fab 3F52 was shown to depend on the type of agonist ligand; orthosteric or allosteric. Our data are compatible with a tilted conformation of the antibody-bound GLP-1R ECD.

## Results

### MAb 3F52 acts as a selective inhibitor of the GLP-1R

**Figure 1:**
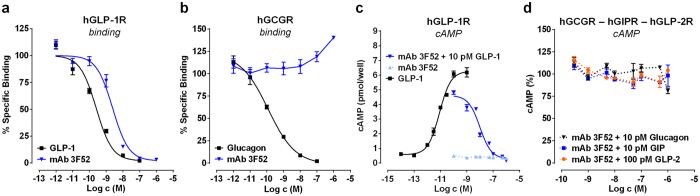
mAb 3F52 selectively inhibits GLP-1 but not glucagon, GIP or GLP-2 receptor. (**a,b**) Receptor radioligand competition binding experiments with membranes of BHK cells stably over-expressing human GLP-1R (**a**) and GCGR (**b**), respectively. (**a**) Displacement of ^125^I-GLP-1 from GLP-1R by GLP-1 and mAb 3F52. (**b**) ^125^I-glucagon is displaced from GCGR by glucagon but not mAb 3F52. (**c,d**) Cell-based cAMP Flashplate^®^ assay with BHK cells stably over-expressing human GLP-1R (**c**), GCGR, GIPR and GLP-2R (**d**). (**c**) GLP-1 induces concentration-dependent increase of intracellular cAMP, and this can be inhibited by mAb 3F52 in a concentration-dependent manner, whereas mAb 3F52 alone is completely inactive. (**d**) MAb 3F52 does not reveal any influence on GCGR-, GIPR- and GLP-2R-mediated cAMP accumulation induced by EC_80_ concentration of the respective ligands. Values have been normalized to the response of the corresponding ligand (GCG, GIP, GLP-2) in the absence of mAb 3F52. Depicted values are mean ± SEM, and are representative of at least two independent experiments.

The binding properties of mAb 3F52 to GLP-1R were characterized in a competition binding assay using ^125^I-GLP-1(7-37)-OH as the tracer on cell membranes expressing the human GLP-1R. The results showed that mAb 3F52 prevented binding of ^125^I-GLP-1 to human GLP-1R in a concentration-dependent manner with an IC_50_ value of ~3 nM (Fig. 1a). The selectivity of mAb 3F52 was further investigated by binding studies of the homologous human receptors for glucagon, GIP and GLP-2. Binding of mAb 3F52 to the human glucagon receptor was characterized in a competition binding assay using ^125^I-glucagon as tracer binding to human glucagon receptor (Fig. 1b). MAb 3F52 did not prevent ^125^I-glucagon from binding to the glucagon receptor. Additionally, mAb 3F52 was characterized for its effect on GLP-1R in a cell-based cAMP accumulation assay. At human GLP-1R, mAb 3F52 prevented GLP-1-mediated activation in a concentration-dependent manner with an IC_50_ of ~10 nM (Fig. 1c). Furthermore, mAb 3F52 functioned as a neutral antagonist, since no agonist activity was detectable for mAb 3F52 alone up to a concentration of 1 μM (Fig. 1c). The inhibitory effect of mAb 3F52 was shown to be selective for GLP-1R as no significant inhibition was observed when the antibody was tested on glucagon-, GIP- or GLP-2-induced cAMP accumulation on their respective receptors (Fig. 1d).

### Fab 3F52 specifically binds to human and cynomolgus monkey GLP-1R

**Figure 2:**
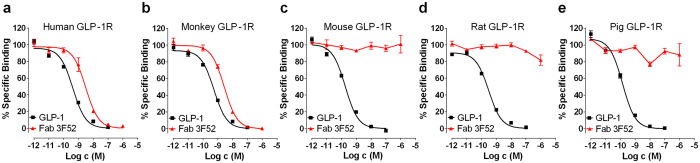
Characterization of Fab 3F52 by ^125^I-GLP-1 competition binding of GLP-1R from different species. (**a–e**) Membranes of BHK cells stably over-expressing GLP-1R specifically bind to ^125^I-GLP-1, which can be displaced by native GLP-1 at all tested receptor species, whereas Fab 3F52 selectively reveals displacement only at human (**a**) and cynomolgus monkey (**b**) GLP-1R, and no competition is recorded at mouse (**c**), rat (**d**) and pig (**e**) GLP-1R. Depicted values are mean ± SEM and are representative of at least two independent experiments.

Antibody Fab fragments are useful crystallization tools and Fab 3F52 was cloned for co-crystallization with the GLP-1R ECD. Initially Fab 3F52 was characterized in radioligand competition binding experiments using GLP-1R from various species in order to confirm the species specificity of mAb 3F52 shown previously by immunohistochemistry^25^. The results revealed that Fab 3F52 prevented binding of ^125^I-GLP-1 to human GLP-1R in a concentration-dependent manner with an IC_50_ value of ~3 nM (Fig. 2a), which is comparable to the affinity showed for mAb 3F52 (Fig. 1a) and superior to the peptide antagonist exendin (9-39)^30^. Similar results were obtained with cynomolgus monkey GLP-1R (Fig. 2b), whereas Fab 3F52 did not prevent GLP-1 binding at mouse, rat and pig GLP-1R (Fig. 2c–e). This result fully agreed with the primate specificity previously shown by immunohistochemistry^25^.

### Crystal structure of Fab 3F52 in complex with the human GLP-1R ECD

**Figure 3:**
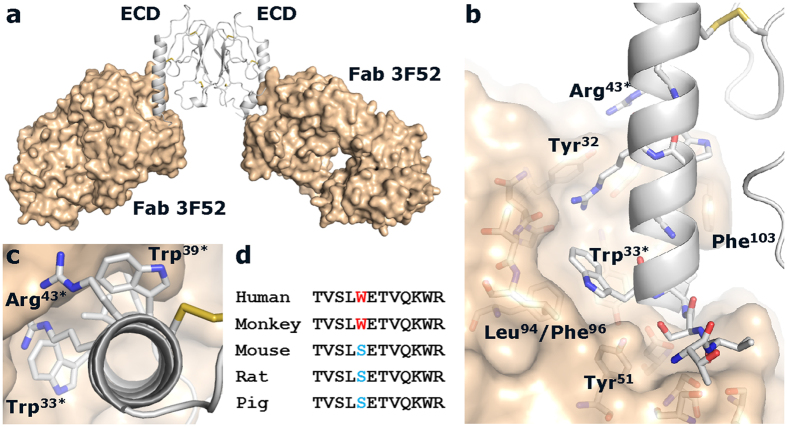
Crystal structure of the GLP-1R ECD/Fab 3F52 complex. The Fab 3F52/ECD complex was crystallized with two complexes in the asymmetric unit as shown in (**a**). The GLP-1R ECD is shown in grey as a ribbon illustration and Fab 3F52 is shown in gold as a surface illustration. (**b**) Zoom in on the Fab-receptor interface showing selected residues at the receptor/Fab interface in sticks. (**c**) Top-down view of the α-helix of the GLP-1R ECD. (**d**) Sequence alignment of the Fab 3F52 epitope of GLP-1R from different species.

The complex between Fab 3F52 and the human GLP-1R ECD was purified by size exclusion chromatography and crystallized in spacegroup P1 with two complexes in the asymmetric unit (Fig. 3a). The structure was determined by molecular replacement and refined to a resolution of 2.0 Å (Table 1). The two complexes in the asymmetric unit are almost identical, except for the most C-terminal part of GLP-1R ECD (Ser^136^-Ile^147^) which is described in more details below. The overall structure of the GLP-1R ECD is essentially unchanged compared to the peptide-bound structures (pdb codes: 3IOL and 3C5T), except for changes of certain sidechain conformations. The GLP-1R epitope of Fab 3F52 involves almost exclusively the α-helix of the ECD; Leu^32^-Arg^43^ (Fig. 3b). The paratope of Fab 3F52 involves residues of all complementarity determining regions (CDRs) of both heavy and light chain variable regions (VH and VL), although the CDR3 loops appear to be responsible for the most critical interactions covering a major part of the hydrophobic interface.

The following interactions describe in details the Fab-receptor interface (GLP-1R residues are designated with ^*^, numbering according to the primary sequences). The backbone of Val^30*^ interacts through hydrogen bonds with the hydroxyl group of Ser^53^, Ser^55^ and Ser^57^ (CDR2 VH). The hydroxyl group of Ser^31^^*^ interacts through a hydrogen bond with the hydroxyl group of Tyr^51^ (CDR2 VH). Leu^32*^ occupies a hydrophobic cleft formed by Tyr^33^, Thr^100^, Ala^101^, Phe^103^ and Tyr^108^ (CDR1 VH and CDR3 VH). Trp^33*^ is a key residue of the GLP-1R epitope and its hydrophobic binding site is defined by Tyr^51^, Leu^94^, Phe^96^ and Tyr^108^ (CDR2 VH, CDR3 VL and CDR3 VH) (Fig. 3b). In addition the backbone nitrogen of Leu^32*^ and Trp^33*^ interact through hydrogen bonds with Asp^34^ (CDR1 VH). The methyl group of Thr^35*^ interacts with Phe^103^ (CDR3 VH). Val^36*^ is also very central in the hydrophobic Fab-receptor interface and interacts with Ala^101^, Phe^103^ and Tyr^108^ (CDR3 VH). Trp^39*^ forms hydrophobic interactions with Phe^103^ and Gly^106^ (CDR3 VH). The guanidine group of Argh^40*^ interacts through hydrogen bonds with the backbone oxygen of Gly^91^, Asp^92^ and Gly^106^ (CDR3 VL CDR3 VH). In addition Arg^40*^ interacts with Tyr^32^ (CDR1 VL) through a cation-π effect. Arg^43*^ forms water-mediated hydrogen bond interactions with Tyr^32^ and Tyr^50^ (CDR1 VL and CDR2 VL).

### Overlapping binding sites of GLP-1 and Fab 3F52

**Figure 4:**
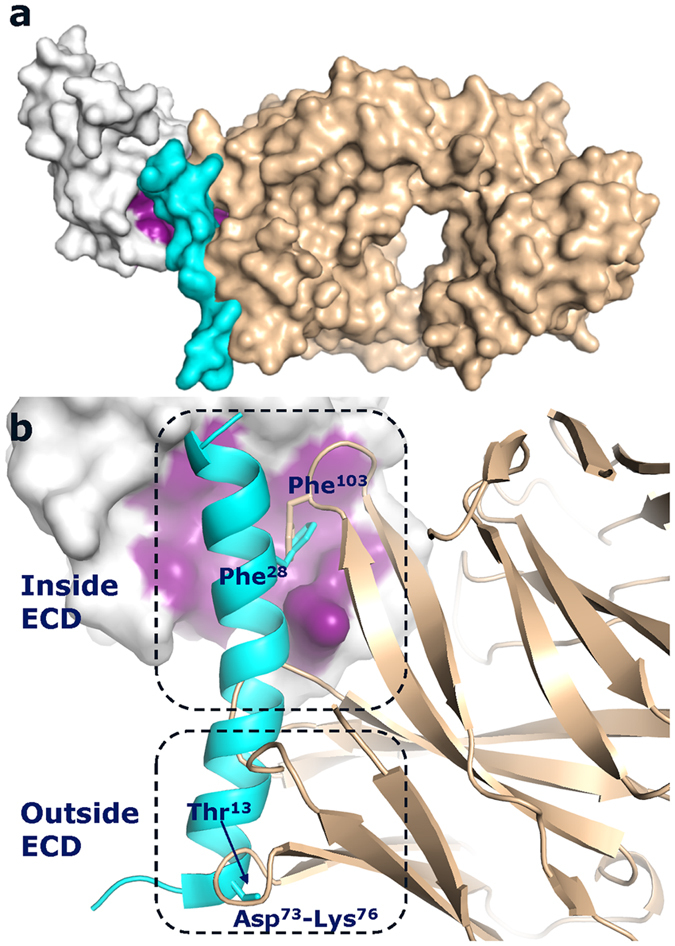
Overlap of GLP-1 and Fab 3F52 binding. (**a**) Superposition of the GLP-1/ECD complex (pdb entry 3IOL) and the Fab 3F52/ECD complex (pdb entry 5E94, this study) using the ECD for the structural alignment. The GLP-1R ECD is shown in grey as a surface illustration; the GLP-1 binding site is highlighted in purple, and GLP-1 and Fab 3F52 are shown as ribbon illustration in cyan and gold respectively. (**b**) Zoom in on the overlapping regions of GLP-1 and Fab 3F52. The area inside and outside the ECD binding site are shown in boxes. Phe^103^ of Fab 3F52 overlaps Phe^28^ of GLP-1. Asp^73^-Lys^76^ of Fab 3F52 overlaps Thr^13^ of GLP-1.

The α-helix of the GLP-1R ECD is a critical part of the GLP-1 binding site as shown previously by x-ray crystallography and it is also the primary binding site of Fab 3F52 as shown in this study^31^. The inhibition of GLP-1 binding by Fab 3F52 is not surprising, considering the degree of overlap between their binding sites (Fig. 4a). The hydrophobic binding site of GLP-1R ECD is occupied by hydrophobic residues of both GLP-1 and Fab 3F52. Phe^28^ of GLP-1 is critical for binding of GLP-1 to GLP-1R and interacts with Leu^32*^, Thr^35^*, Val^36^* and Trp^39^*. Phe^103^ of Fab 3F52 interacts with the same residues of GLP-1R but has a different orientation in the binding site compared to Phe^28^ of GLP-1 (Fig. 4b). Interestingly, the physical overlap of GLP-1 and Fab 3F52 extends outside the binding site of the GLP-1R ECD as illustrated on Fig. 4b. The following regions of the Fab 3F52 heavy chain overlap the entire N-terminal half of the GLP-1 α-helix (Thr^13^-Ala^24^): the tip of CDR2 (Ser^53^-Ser^55^), part of CDR1 (Ser^31^-Tyr^33^), Ile^29^-Thr^30^ next to CDR1 and a framework 3 (FR3) loop defined by Asp^73^-Lys^76^. To our knowledge this is the most extensive overlap described of a Class B GPCR antibody and peptide agonist.

### Fab 3F52-mediated GLP-1R inhibition is agonist type-dependent

**Figure 5:**
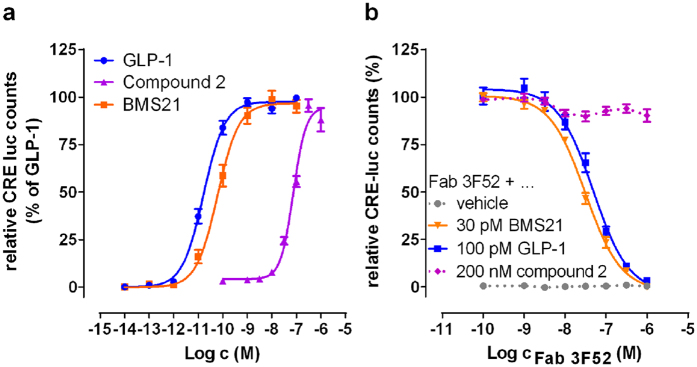
Fab 3F52 inhibits GLP-1R activation mediated by the short GLP-1 analogue BMS21 but not by the small molecule compound 2. (**a,b**) Functional analyses by use of CRE-luc reporter gene assay and BHK cells stably over-expressing human GLP-1R and cAMP response element (CRE) coupled to firefly luciferase (CRE luc). (**a**) Concentration-response curves induced by native GLP-1, the short GLP-1 analogue BMS21 and the small molecule compound 2. Data are normalized to maximum response of GLP-1. (**b**) Influence of increasing Fab 3F52 concentrations on CRE-luc activity triggered by stimulation with EC_80_ of native GLP-1, BMS21 and compound 2. Fab 3F52 reveals concentration-dependent inhibition of GLP-1 and BMS21, but not compound 2. (**a,b**) Depicted values represent mean ± SEM of at least three independent experiments.

The structural overlap between Fab 3F52 and the N-terminal half of GLP-1 inspired further characterization of the inhibition mode of Fab 3F52. The influence of Fab 3F52 on a short GLP-1 analogue BMS21 as well as on the ago-allosteric small-molecule compound 2 was evaluated^18^,^32^. Initially, the agonist properties of these ligands were compared to GLP-1 in a cAMP-sensitive luciferase reporter gene assay (CRE-luc) and revealed potency as reported previously (Fig. 5a). The inhibitory properties of Fab 3F52 were investigated by adding increasing concentrations of Fab 3F52 in the presence of constant EC_80_ concentrations of the three individual agonists (Fig. 5b). Interestingly, Fab 3F52 blocked the BMS21-induced response with potency in the low nanomolar range, similar to the inhibition of native GLP-1. Furthermore, the GLP-1R activity induced by the ago-allosteric small-molecule compound 2 was not significantly influenced by Fab 3F52 and Fab 3F52 did not trigger any receptor activity on its own, as also observed with mAb 3F52 (Fig. 5b). These results showed that the inhibitory effect of Fab 3F52 depends on the type of agonist. Fab 3F52 acts as a neutral antagonist on orthosteric ligands such as native GLP-1 and BMS21, but not on the ago-allosteric small molecule compound 2.

### Fab 3F52 acts as competitive GLP-1R antagonist

**Figure 6:**
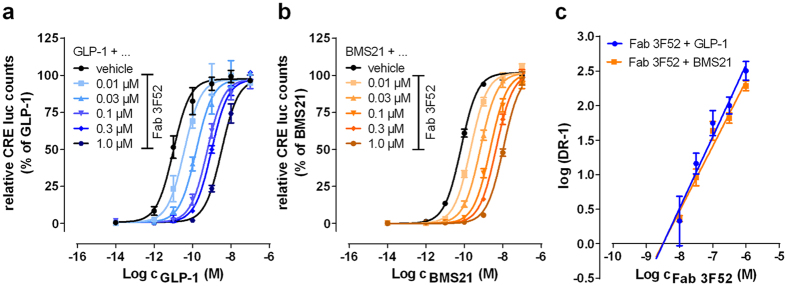
Fab 3F52 reveals competitive interactions with native GLP-1 and BMS21. Concentration-response curves for native GLP-1 (**a**) and the short GLP-1 analogue BMS21 (**b**) in the absence and presence of increasing Fab 3F52 concentrations, obtained by stimulation of BHK cells stably over-expressing human GLP-1R and cAMP response element (CRE) coupled to firefly luciferase (CRE luc). Data are normalized to maximum response of GLP-1 (**a**) and BMS21 (**b**), respectively, in the absence of Fab 3F52. (**c**) Schild regression analyses of concentration-response curves depicted in (**a**,**b**). Dose ratio (DR) is the ratio of the apparent EC_50_ in the presence of a given Fab 3F52 concentration over the EC_50_ in the absence of Fab 3F52. The Schild plot intersects the abscissa at pA2. (**a–c**) Values shown represent mean ± SEM of at least 3 independent experiments.

We aimed to clarify whether the Fab 3F52-mediated inhibition of BMS21 occurred in a competitive manner. Agonist concentration-response curves of native GLP-1 and BMS21 were monitored in the absence and presence of increasing Fab 3F52 concentrations using the CRE-luc reporter gene assay (Fig. 6a,b) and Schild regression analyses were performed based on the determined EC_50_ values (Fig. 6c). As expected from the binding results and the crystal structure shown in this report, GLP-1 concentration-response curves were displaced with no depression of maxima (Fig. 6a) and Schild regression analysis of these shifts revealed a unit slope confirming a competitive mode of action (Fig. 6c, Table 2). Interestingly, similar results were obtained with BMS21 (Fig. 6b,c). Fab 3F52 induced parallel rightward shifts, resulting in pA2 of ~8.5 for both tested peptides (Table 2). These results suggested that Fab 3F52 acted as a competitive antagonist on both native GLP-1 and on the short GLP-1 analogue BMS21.

## Discussion

The α-helix of the GLP-1R ECD is important for ligand binding and specificity of GLP-1R 31,33. Binding of the small molecule antagonist T-0623 to human but not rodent GLP-1R is determined by Trp^34^ 34. In this study, the crystal structure of the GLP-1R ECD/Fab 3F52 complex showed that Trp^33^ is a key residue at the Fab-receptor interface. Apparently, Trp^33^ is accessible for binding of both small and large molecules which is interesting considering the spatial relationship between the ECD and TM domain of GLP-1R. Trp^33^ is also the only divergent residue in the Fab 3F52 epitope of the different GLP-1R species suggesting that Trp^33^ is the sole determinant of Fab 3F52 species specificity. The epitope sequence alignment in Fig. 3d shows that Trp^33^ is conserved in human and monkey GLP-1R whereas mouse, rat and pig have a serine residue in the corresponding position. Furthermore, the homologous receptors for glucagon, GIP and GLP-2 all lack this particular tryptophan residue, which explains the lack of mAb 3F52 binding to these receptors.

In previous structures of the GLP-1R ECD, the C-terminal part (Ser^129^-Ile^147^), which links the ECD with TM1 in the full length receptor, was not described because of flexibility. In the Fab 3F52 complex presented here, the Ser^129^-Ser^135^ segment remained flexible but the most C-terminal segment Glu^138^-Phe^143^ formed an α-helix. The Glu^138^-Phe^143^ α-helix was observed in only one of the two complexes in the asymmetric unit and its B-factors were high compared to the critical epitope of the ECD, suggesting that it was not tightly bound to the Fab and maybe not relevant at all. However, the Glu^138^-Phe^143^ sequence clearly has α-helical propensity, which is in agreement with the recent crystal structure of the homologous glucagon receptor TM domain showing an extracellular α-helical extension of TM1^20^. The TM1 α-helix of GLP-1R may extend out of the membrane at least until Pro^137^, in a manner similar to the glucagon receptor.

Fab 3F52 blocks the GLP-1 binding site of the GLP-1R ECD directly as depicted in Fig. 4, which is sufficient to explain the inhibitory effect on GLP-1-mediated activation of GLP-1R. However, the structure of the Fab 3F52/ECD complex revealed a broader overlap between the two ligands which extends outside the ECD binding site. In fact Fab 3F52 overlaps the entire N-terminal half of the GLP-1 α-helix which is expected to approach the TM domain of GLP-1R according to the two-domain binding model of Class B GPCRs. The short GLP-1 analogue BMS21 corresponds in length approximately to the first 11 residues of GLP-1 (His^7^-Ser^17^), including at least 5 residues (Thr^13^-Ser^17^) that overlap with Fab 3F52 according to the structure presented here. We believe that GLP-1 and BMS21 share the same binding site in the GLP-1R TM domain given their high sequence identity (7 identical of out 11 residues). Moreover previous results suggested that BMS21 does not engage the orthosteric binding site of the ECD and we showed that a closely related analogue of BMS21 does not interact with the isolated ECD (see Supplementary Fig. S1)^35^. The new structural information about the GLP-1R ECD/Fab 3F52 complex was linked with effects on the full length GLP-1R, by performing a pharmacological evaluation of the inhibitory properties of Fab 3F52. Interestingly, Fab 3F52 inhibited activation by BMS21 and the Schild regression analysis indicated a competitive mechanism of Fab 3F52. The physical overlap of the two ligands outside the ECD binding site shown in Fig. 4b, corroborates the competitive mechanism of Fab 3F52, suggesting that Fab 3F52 inhibits BMS21 by a direct effect upon binding to GLP-1R. In particular the Asp^73^-Lys^76^ loop of Fab 3F52 overlaps with Thr^13^ only 6 residues away from the N-terminal histidine (His^7^) of GLP-1 which is critical for receptor activation.

The ago-allosteric small molecule compound 2 enhances binding of the agonist GLP-1 but does not affect binding of the antagonist exendin (9-39) and it cannot be inhibited by the peptide antagonist exendin (9-39), which is not surprising considering the intracellular binding site of compound 2^32^,^35^,^36^. Likewise, Fab 3F52 was unable to inhibit compound 2-mediated activation of GLP-1R, suggesting that binding of Fab 3F52 to GLP-1R does not lead to a general stabilization of GLP-1R in an in-active conformation. These data also support the conclusion that Fab 3F52 directly blocked binding of BMS21, implying that Fab 3F52 occupies partially the orthosteric binding site of the GLP-1R TM domain, which is interesting considering the spatial relationship of the ECD, the antibody and the TM domain. In particular we are intrigued by the accessibility of a large protein such as Fab 3F52 into a region of GLP-1R normally accessed by the activation determinants of GLP-1.

Glucagon and GLP-1 are homologous peptides that activate homologous receptors and moreover glucagon cross-reacts on GLP-1R with full efficacy but reduced potency compared to GLP-1^37^,^38^. The close relationship between these two receptors was highlighted previously by showing that glucagon had full potency and full efficacy on a chimeric receptor containing the ECD of GCGR and the TM domain of GLP-1R^38^. Based on this observation it would seem likely that both the apo-forms and the peptide agonist-bound forms of these receptors are quite similar.

**Figure 7:**
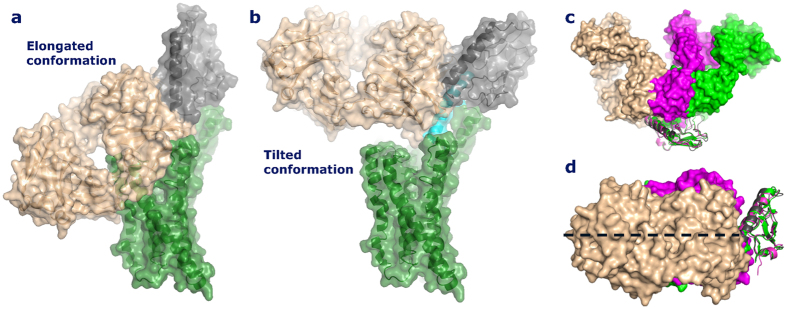
Interpretation of glucagon-family Class B GPCR crystal structures. (**a,b**) The Figures illustrate elongated and tilted conformations of the Fab 3F52-bound GLP-1R ECD in the context of the homologous GCGR TM domain. The illustrations are based on the crystal structures of the GCGR TM domain, the Fab 3F52/GLP-1R ECD complex and the GLP-1/GLP-1R ECD complex (pdb entry 4L6R, 5E94 and 3IOL, respectively). The BRIL fusion partner was removed from the coordinates of 4L6R and the C-terminus of the GLP-1R ECD (Glu^128^) was positioned to enable the link with TM1 of GCGR (Met^123^). The GLP-1R ECD is shown in dark grey, Fab 3F52 is shown in gold, GLP-1 is shown in cyan and the GCGR TM domain is shown in green. (**a**) An elongated conformation of the Fab-bound GLP-1R ECD is not possible due to collision with the TM domain and the membrane. (**b**) A tilted conformation of the ECD is possible where Fab 3F52 occupies partly the orthosteric binding site in the TM domain. (**c,d**) Structural alignment of the ECD of the three class B GPCR ECD/Fab complexes; GIP (magenta, pdb entry 4HJ0), glucagon (green, pdb entry 4ERS) and GLP-1 (golden/grey, pdb entry 5E94). (**c**) This view illustrates the differences in the binding mode of the three Fab fragments. (**d**) This view shows the similarity of the Fab binding modes. The angle between the long axis of the Fab fragments (broken line) and the axis defined by the α-helix of the ECD is almost identical in the three structures.

In the elongated conformation of the antibody-bound GCGR the α-helix of the ECD was roughly perpendicular to the plane of the membrane^22^. It is clear that in the Fab 3F52-bound conformation of the full length GLP-1R, the N-terminal α-helix of the ECD is not perpendicular to the plane of the membrane, because the Fab fragment would collide with the membrane and the TM domain (Fig. 7a). Instead the Fab 3F52/ECD structure is compatible with a tilted conformation of the ECD, where Fab 3F52 occupies partially the orthosteric binding site of the GLP-1R TM domain explaining the competitive effect of Fab 3F52 on BMS21-induced activation of GLP-1R (Fig. 7b). Alternatively Fab 3F52 may inhibit BMS21 by an indirect mechanism, although less likely considering the competitive mode of action indicated by the Schild plot regression analysis (Fig. 6c). The apo-form of the GCGR receptor may exist in a closed state as shown by dynamic simulation where the α-helix of the ECD is parallel to the plane of the membrane and a conformational change is required to adopt the open glucagon-bound state of GCGR^22^. Assuming similar structural properties of GLP-1R and GCGR, binding of Fab 3F52 to a closed state of GLP-1R may prevent a conformational change of the ECD relative to the TM domain required for BMS21-induced activation of GLP-1R. Nevertheless it seems that the Fab-bound conformations of GCGR and GLP-1R are different; GCGR adopts an elongated conformation whereas GLP-1R adopts a tilted or closed conformation. It is important to note that the Fab-bound conformations may be different from the peptide agonist-bound conformations and that the ligand-bound forms may be different from the apo-form as suggested previously for GCGR^22^. An elongated conformation may be compatible with binding of GLP-1 similar to the model of glucagon-bound GCGR^22^.

The structures of three homologous Class B GPCR ECD/Fab complexes have been solved by x-ray crystalloragphy: GIP, glucagon and GLP-1 (in the present study)^28^,^29^. All three Fab fragments are competitive antagonists and block the natural hormone binding site of the ECD directly. There are differences in their binding mode as shown in Fig. 7c; however this study also revealed an interesting similarity as the angle between the long axis of the Fab fragments and the axis defined by the α-helix of the ECD is almost identical in the three structures (Fig. 7d). This similarity results from the screening process as all three antibodies were selected based on their ability to block peptide agonist binding and/or activation of the full length receptor on whole cells. In this context the accessible antibody epitopes of the ECD were defined by the proximity with the membrane and by the orientation of the ECD relative to the membrane. In addition, the proximity between the ECD and the TM domain limits the accessibility of an antibody for binding to the ECD. The identification of antibodies with a similar binding angle suggests the existence of a common structural feature, maybe a common apo-form or a common antibody-bound conformation. Of note, none of the Fab fragments collide with the membrane if the ECD assumes either a tilted or closed conformation.

This study provides a clear explanation of the primate specificity of mAb 3F52 demonstrated by receptor binding and x-ray crystallography. MAb 3F52 is to our knowledge the best characterized monoclonal antibody for specific identification of primate GLP-1R in cells and tissues. The structure-function relationship established in this study provides new insight regarding the antibody inhibitory mode of action and the overall conformation of the antibody-bound GLP-1 receptor. High resolution structure determination is necessary to fully clarify the molecular details of peptide- and antibody-bound class B GPCRs, which is important for the development of new and improved therapeutics targeting this class of GPCRs.

## Methods

### Materials and reagents

Tissue culture media and reagents were purchased from Life Technologies. Native GLP-1(7-37)-OH, glucagon, BMS21, compound 2, ^125^I-GLP-1 and ^125^I-glucagon tracer were synthesized by Novo Nordisk A/S. GIP and GLP-2 were purchased from Bachem. All other laboratory reagents were obtained from Sigma-Aldrich unless otherwise specified. BMS21 was assembled on rink-amide (0.57 mmol/g) at a 0.25 mmol scale using manual Fmoc chemistry. Fmoc amino acids were purchased from Protein Technologies or Novabiochem, Fmoc-(S)-2′-methyl Biphenylalanine, Fmoc-(S)-2′-ethyl-4′-methoxy biphenylalanine and Fmoc-(S)-2-fluoro-R-methylphenylalanine were synthesized as described previuosly^18^. Loading of Fmoc-(S)-2′-methyl Biphenylalanine to the Rink amide resin was conducted using 5 eq aminoacid preactivated with N,N’-Diisopropylcarbodiimide, 1-Hydroxy-7-azabenzotriazole for 30 minutes and coupled to the resin overnight. The following aminoacids were coupled using 4 eq Fmoc amino acids preactivated with 1-Ethyl-3-(3-dimethylaminopropyl)carbodiimide and 1-Hydroxy-7-azabenzotriazole for 30 minutes followed by coupling for 2 hours. Cleavage and purification was done similar described previously^18^.

### Cloning and sequencing of mAb 3F52

Coding sequences spanning the VH and VL regions of the murine anti-GLP-1R antibody, mAb 3F52 were obtained from hybridoma cell RNA. VH and VL sequences were amplified using the SMART™ RACE cDNA amplification kit (Clontech). The SMART™ RACE universal primer mix (UPM) was used as forward primer in combination with either a reverse primer specific for the murine heavy chain constant region (CH) (5′-CCCTTGACCAGGCATCCCAG-3′) or a reverse primer specific for the murine kappa type light chain constant region (CL) (5′-GCTCTAGACTAACACTCATTCCTGTTGAAGCTCTTG-3′). PCR products were isolated and cloned for DNA sequencing using a Zero Blunt® TOPO® PCR Cloning Kit and chemically competent TOP10 *E.coli* cells (Life Technologies). All kits and reagents were used according to the manufacturer’s instructions.

### Vectors for transient expression of a 3F52 Fab fragment

CMV promotor-based LC and HC expression vectors (pTT vectors) were generated for transient expression of a mouse/human chimeric Fab fragment of mAb 3F52. The pTT vectors were developed for transient protein expression in HEK293-6E cells by Yves Durocher. The 3F52 VL and VH regions were PCR amplified and cloned by standard restriction-based cloning into a toolbox vectors carrying a human kappa light chain CL or a truncated human IgG4 CH, respectively. The human CH sequence was truncated in the hinge region after the IgG4 hinge lysine. The cloning reaction was subsequently transformed into *E.coli* TOP10 cells (Life Technologies) for selection. The sequence of the final construct was verified by DNA sequencing.

### Expression and purification of Fab 3F52

The recombinant Fab 3F52 was expressed in human embryonic kidney suspension cells HEK293-6E^39^. The cells were transiently co-transfected with the expression vectors encoding heavy chain and light chain using 293fectin^TM^ (Invitrogen) in serum-free FreeStyle™ 293 Expression Medium (Gibco). Supernatant was harvested five days post-transfection, filtered and applied to a KappaSelect affinity column (GE Healthcare). The bound Fab was eluted with 25 mM sodium citrate, pH 3.2 and pooled for further purification by size-exclusion chromatography on a Superdex75 column (GE Healthcare) in phosphate buffered saline (PBS). Purified protein was sterilized by filtration through a 0.2 mm filter unit (Sartorius). The purity and identity of final Fab 3F52 was analysed by SDS-PAGE, size-exclusion HPLC and mass spectrometry.

### Cell culture

Baby hamster kidney (BHK) cells stably over-expressing human, cynomolgus, rat, mouse and pig GLP-1 receptor, human glucagon receptor, human GIP receptor, human GLP-2 receptor or co-expressing hGLP-1R and cAMP response element (CRE) coupled to firefly luciferase (CRE luciferase) were generated at Novo Nordisk A/S. The cells were grown at 37 °C and 5% CO_2_ in DMEM supplemented with 100 IU/mL penicillin, 100 μL/mL streptomycin, 10% fetal calf serum and 1 mg/mL geneticin G-418. Cell medium for the double stable CRE luc BHK cell line additionally contained 240 nM methotrexate.

### Receptor radioligand competition binding

The binding properties of mAb 3F52 and Fab 3F52, respectively, were tested by Scintillation Proximity Assay (SPA)(Perkin Elmer) on membranes of BHK cells stably over-expressing human, cynomolgus, rat, mouse and pig GLP-1 receptors. For plasma membrane preparation, cells were homogenized by the Ultrathurax in buffer (20 mM Na-HEPES, 0.1 mM EDTA (pH = 7.4)) and stored at −80 °C until usage. For binding analyses, membranes were diluted in assay buffer (50 mM HEPES, 5 mM EGTA, 5 mM MgCl_2_, 0.005% Tween 20, pH = 7.4) to a final concentration of 0.2 mg/ml of protein and distributed (3 μg/well) to 96-well OptiPlates. Afterwards, membranes were incubated 2 hours at 30 °C in the presence of SPA beads (0.5 mg/well), 0.06 nM [^125^I]GLP-1 and mAb 3F52 / Fab 3F52 in increasing concentrations. After incubation, SPA beads were pelleted by centrifugation (10 minutes, 1500 rpm) and counted in a TopCount (Perkin Elmer). GCGR binding experiments with mAb 3F52 were performed in the same procedure, but with membranes of BHK cells stably over-expressing glucagon receptor and ^125^I-glucagon (0.06 nM).

### cAMP accumulation assay

Modulation of the intracellular second messenger cAMP was analysed by use of Adenylyl Cyclase Activation Flashplate^®^ Assay in whole cells stably over-expressing the respective receptor (BHK-hGLP-1R, BHK-hGCGR, BHK-hGIPR, BHK-hGLP-2R) according to manufacturer’s instructions (Perkin Elmer). In short, mAb 3F52 was diluted in assay buffer (HBSS plus 20 mM Hepes and 0.1% Pluronic F-68) and added to the wells of the 96-well FlashPlate (25 μl). Then cells were suspended in Stimulation Buffer (provided with the assay kit), added to the plate (120,000 cells/50 μl/well), shaken 5 minutes at room temperature (RT) and then incubated 25 minutes at RT in the absence or presence of mAb 3F52. Afterwards receptor agonists (GLP-1, GIP, GCG, GLP-2), diluted in assay buffer, were transferred to cell suspension, shaken 5 minutes at RT followed by 25 minutes incubation at RT. Reaction was finished by addition of detection mix (provided with the assay kit), plates were mixed for 30 minutes and then rested for three hours at RT before counting in a TopCount (Perkin Elmer).

### CRE luciferase reporter gene assay

A cAMP-sensitive luciferase reporter gene assay has been applied to further characterize receptor functionality. Therefore BHK cells stably co-expressing human GLP-1R and cAMP response element (CRE) coupled to firefly luciferase (CRE luc) were aliquoted and stored in liquid nitrogen until usage. Before each assay appropriate amount of aliquots were thawed, suspended (1 × 10^5^ cells/ml) in assay medium (DMEM w/o phenol red, 10 mM Hepes, 2% ovalbumin and 0.2% Pluronic F-68) and then transferred into a 96 well plate (5 × 10^3^ cells/50 μl) followed by addition of ligand and/or Fab 3F52 diluted in assay medium (50 μl/well). When influence of Fab 3F52 on GLP-1R ligands (native GLP-1, BMS21, compound 2) was examined, ligands plus Fab 3F52 were added simultaneously to the cells (50 μl/well). The assay plate was incubated for 3 hours in a 5% CO_2_ incubator at 37 °C and then rested at RT for 15 minutes before steadylite plus reagent (Perkin Elmer) was added to each well of the assay plate (100 μl/well) followed by 30 minutes slowly shaking at RT protected from light. Finally the plates were read in a Synergy Multimode reader (BioTek).

### Data analysis of *in vitro* pharmacology experiments

Nonlinear regression and Schild plot analyses were performed with GraphPad Prism 6 (GraphPad Software, San Diego).

### Crystallization and structure determination

Expression, refolding and purification of GLP-1R ECD were performed as previously described^30^. GLP-1R ECD and Fab 3F52 was mixed 1:1 and the complex was purified by size exclusion chromatography. Single crystals were obtained using the JCSG screen (Molecular Dimensions) condition H3: 0.2 M Sodium dihydrogen Phosphate monohydrate, 20% w/v PEG 3350. Diffraction data was collected at the MaxLab 911-3 synchrotron, Lund University Sweden and processed using HKL2000^40^. The structure was solved by molecular replacement using the extracellular domain of the GLP1 receptor (PDB code 3IOL) and a homology model of Fab 3F52 manually constructed using the coordinates of an homologous Fab (PDB entry 1BZ7) as search models and subsequently refined. All structure calculations were performed using programs of the Phenix suite, including manual model building in Coot^41^,^42^. The coordinates and structure factors were deposited in the Protein Data Bank; PDB entry 5E94. Structure figures were made in PyMol (The PyMOL Molecular Graphics System, Version 1.7.0.3 Schrödinger, LLC).

## Additional Information

**How to cite this article**: Hennen, S. *et al*. Structural insight into antibody-mediated antagonism of the Glucagon-like peptide-1 Receptor. *Sci. Rep.* **6**, 26236; doi: 10.1038/srep26236 (2016).

## Supplementary Material

Supplementary Information

## Figures and Tables

**Table 1:** Data collection and refinement statistics.

Diffractions statistics	
Wavelength [Å]	1.0000
Resolution range [Å]	32.0–2.0 (2.07–2.0)
Space group	P 1
Unit cell parameters [Å, °]	a = 57.20 b = 65.69 c = 88.35α = 111.61 β = 97.47 γ = 91.28
No. of observations	121323 (12226)
No. of unique reflections	72374 (7448)
Multiplicity	1.7 (1.6)
Completeness	0.90 (0.95)
Mean I/σ(I)	13.9 (3.8)
Wilson B-factor [Å^2^]	27.28
R_merge_	0.039 (0.225)
R_meas_	0.055 (0.318)
CC_1/2_	0.998 (0.858)
CC*	0.999 (0.961)
Refinement	
No. of reflections used in refinement	72235 (7445)
No. of reflections used for R_free_	3616 (381)
R_work_	0.188 (0.214)
R_free_	0.232 (0.264)
Number of non-hydrogen atoms	8974
macromolecules	8507
No. of protein residues	1086
Bond r.m.s.d [Å]	0.008
Angle r.m.s.d [°]	1.12
Ramachandran favored (%)	97
Ramachandran outliers (%)	0.19
Average B-factor	32.45
macromolecules	32.42
solvent	32.89

Statistics for the highest-resolution shell are shown in parentheses.

**Table 2:** Schild regression analyses.

	Slope	95% CI	pA2	95%CI
GLP-1	1.04	0.76 to 1.32	8.49 (~*3.16* *nM*)	9.08 to 8.14
BMS21	0.94	0.80 to 1.09	8.50 (~*3.24 nM*)	8.80 to 8.28

The values determined by Schild regression analyses (Fig. 6) clearly indicate a competitive mode of action between Fab 3F52 and the two investigated GLP-1R agonists, GLP-1 and BMS21.
