# Supplementary material for: Structural insight into antibody-mediated antagonism of the Glucagon-like peptide-1 Receptor
Source: Sci Rep. 2016 May 19;6:26236. doi: 10.1038/srep26236 (PMC4872540; doi:10.1038/srep26236)
Supplement: Supplementary Information [file srep26236-s1.pdf]

Supplementary Information

Structural insight into antibody-mediated antagonism of the Glucagon-like peptide-1 Receptor

Stephanie Hennen,[1] János T. Kodra,[2] Vladyslav Soroka,[3] Berit O. Krogh,[4] Xiaoai Wu,[5] Peter Kaastrup,[6] Cathrine Ørskov,[1] Sif G. Rønn,[1] Gerd Schluckebier,[3] Silvia Barbateskovic,[3] Prafull S. Gandhi,[7] and Steffen Reedtz-Runge[3].

[1]Incretin Biology, [2]Protein & Peptide Chemistry 3, [3]Protein Structure, [4]Yeast Expression Systems, [6]Antibody Technology, [7]Protein Interaction, Novo Nordisk A/S, Novo Nordisk Park, DK-2760 Måløv, Denmark. [5]Protein Chemistry 1, Novo Nordisk A/S, Novo Nordisk China R&D, Beijing, China.

To whom correspondence should be addressed: Steffen Reedtz-Runge, Protein Structure, Novo Nordisk Park G8S438, Novo Nordisk A/S, DK-2760, Denmark. E-mail: sffr@novonordisk.com. Phone: +4530754431

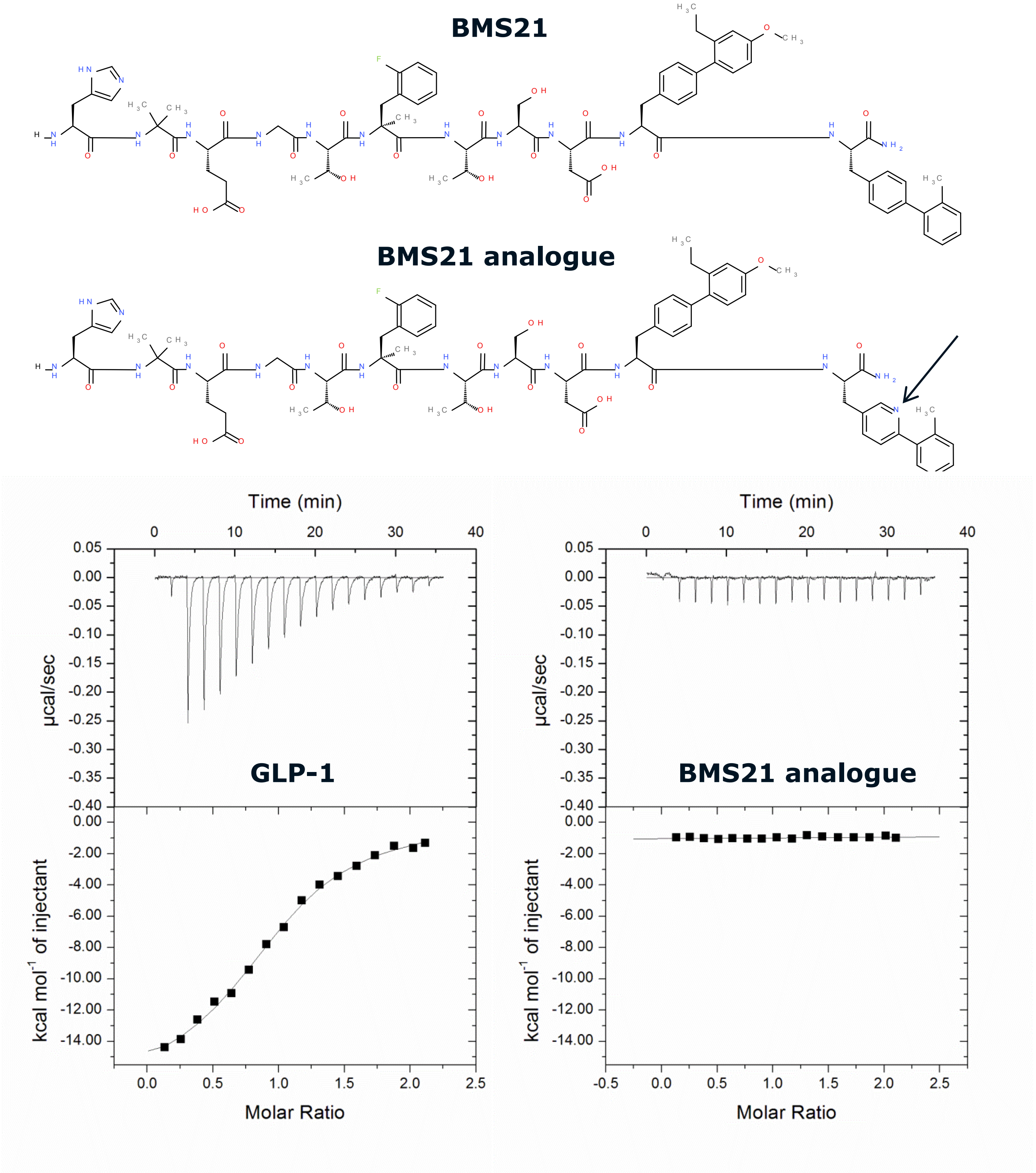

Supplementary Figure S1. The BMS21 analogue does not interact with the isolated N-terminal extracellular domain of the GLP-1 receptor. Binding of GLP-1 and a BMS21 analogue (SEQ ID No.9 in patent WO2006127948) to the isolated GLP-1R ECD was characterized by isothermal titration calorimetry (ITC). BMS21 and the closely related analogue of BMS21 differ by only a single atom as indicated by the arrow and they activate the full length GLP-1R with similar potency (data not shown). The binding parameters of GLP-1 are an average of triplicates showing 1:1 binding stoichiometry and a K_d of 1.3 µM (n = 0.96 ± 0.016, ΔH = -16.2 ± 0.66 kcal/mol, K = 7.39e5 ± 1.82e5 1/M, ΔS = -27.5 ± 2.8 cal/mol/deg). The BMS21 analogue showed no binding.

Supplementary Method. Binding of the GLP-1 and the BMS21 analogue to the isolated GLP-1R ECD was characterized by ITC in 10 mM Tris-HCL buffer pH 7.5, at 25°C, using an iTC200 calorimeter (Malvern). The sample cell contained 12 µM GLP-1R ECD and the concentration of the injected ligands was 120 µM. The heat associated with each injection of ligand was integrated and plotted against the molar ratio of ligand to macromolecule. Thermodynamic parameters were extracted from a curve fit to the data using the software (Origin 7.0) provided by Malvern according to a one-site binding model. Experiments were performed in triplicate with excellent reproducibility (<10% variation in thermodynamic parameters).

Supplementary Reference. Ewing, W.R. et al. N-terminally modified GLP-1 receptor modulators. Patent WO2006127948.
